# Supplementary material for: Emergence of sliding ferroelectricity in naturally parallel-stacked multilayer ReSe2 semiconductor
Source: Nat Commun. 2025 Jul 9;16:6313. doi: 10.1038/s41467-025-61756-4 (PMC12238609; doi:10.1038/s41467-025-61756-4)
Supplement: Supplementary file 1 — Supplementary Information [file 41467_2025_61756_MOESM1_ESM.pdf]

Supplementary Information

Emergence of sliding ferroelectricity in naturally parallel-stacked multilayer $ReSe_2$ semiconductor

Wuhong Xue[1,2,#], Peng Wang[1,2,#], Wenjuan Ci[1,2,#], Ying Guo[3], Jingyuan Qu[1,2], Zeting Zeng[1,2], Tianqi Liu[1,2], Ri He[4*], Shaobo Cheng[3*], Xiaohong Xu[1,2*]

[1]Key Laboratory of Magnetic Molecules and Magnetic Information Materials of Ministry of Education, School of Chemistry and Materials Science, Shanxi Normal University, Taiyuan 030031, China

[2]Research Institute of Materials Science, Shanxi Key Laboratory of Advanced Magnetic Materials and Devices, Shanxi Normal University, Taiyuan 030031, China

[3]Henan Key Laboratory of Diamond Optoelectronic Materials and Devices, Key Laboratory of Material Physics, Ministry of Education, School of Physics and Microelectronics, Zhengzhou University, Zhengzhou 450052, China

[4]Key Laboratory of Magnetic Materials Devices, Zhejiang Province Key Laboratory of Magnetic Materials and Application Technology, Ningbo Institute of Materials Technology and Engineering, Chinese Academy of Sciences, Ningbo 315201, China

[*]Corresponding authors: Ri He, heri@nimte.ac.cn; Shaobo Cheng, chengshaobo@zzu.edu.cn; Xiaohong Xu, xuxh@sxnu.edu.cn

[#]These authors contributed equally: Wuhong Xue, Peng Wang, Wenjuan Ci.

This PDF file includes:

Supplementary

Figs. 1 to 18

References (1-4)

Table of Contents

Fig. S1. Transfer characteristic curve of the $ReSe_2$ field effect transistor.

Fig. S2. Thickness of $ReSe_2$ flakes for UV-Vis-NIR absorption spectra, Raman spectroscopy, and SHG spectroscopy.

Fig. S3. Raman characterization of $ReSe_2$ at different polarization angles.

Fig. S4. SHG characterization of $ReSe_2$ with a 1064 nm laser.

Fig. S5. Atomic configuration diagram of interlayer sliding in bilayer $ReSe_2$.

Fig. S6. Schematic of atomic arrangement of intermediate state in six-layer $ReSe_2$.

Fig. S7. Charge density difference of the sliding one layer in different-layer $ReSe_2$ systems.

Fig. S8. Out-of-plane ferroelectric domains in multilayer $ReSe_2$ flakes.

Fig. S9. Local phase and amplitude hysteresis loops in 2L $ReSe_2$ flake.

Fig. S10. The evolution of out-of-plane amplitude with driving AC voltage in 1T' $ReSe_2$.

Fig. S11. Temperature-dependent SHG intensity in 1T' $ReSe_2$.

Fig. S12. Optical images and nanoflake thicknesses of $ReSe_2$ devices.

Fig. S13. Switchable diode characteristics of $ReSe_2$ device under opposite poling biases.

Fig. S14. Band diagrams of the $ReSe_2$ ferroelectric diode in different polarized states.

Fig. S15. *I–V* curves of $ReSe_2$ flake at different DC biases obtained from C-AFM measurements.

Fig. S16. Surface potential differences of 1T' $ReSe_2$ after applying different poling voltages.

Fig. S17. Optical power density dependence of V_{oc} in different sliding ferroelectric photovoltaic devices.

Fig. S18. Photovoltaic effect under different light intensities and polarization voltages.

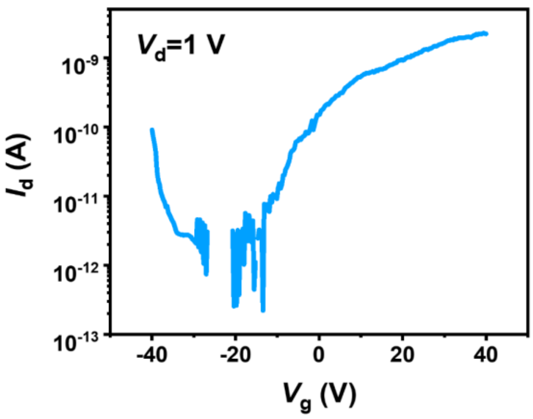

Supplementary Fig. 1 Transfer characteristic curve of the $ReSe_2$ field effect transistor, showing ambipolar semiconductor behavior.

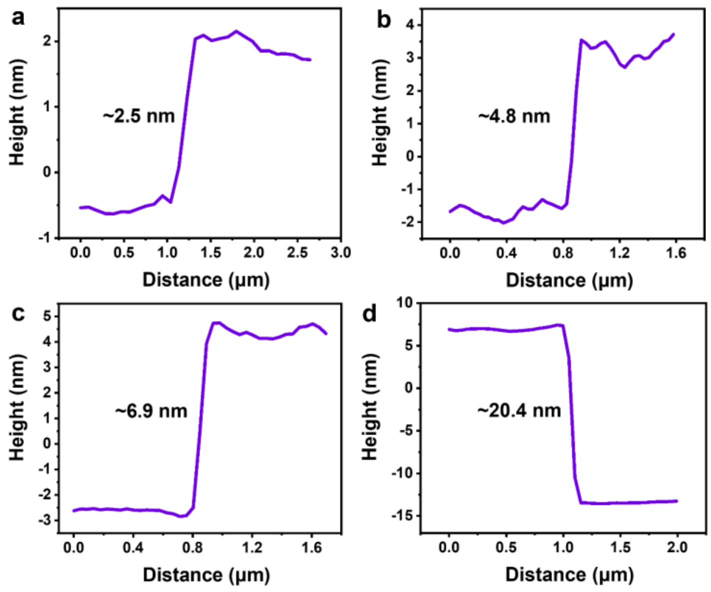

Supplementary Fig. 2 a-d The AFM height profile images of $ReSe_2$ flakes for characterizing UV-Vis-NIR absorption spectra, Raman, and SHG.

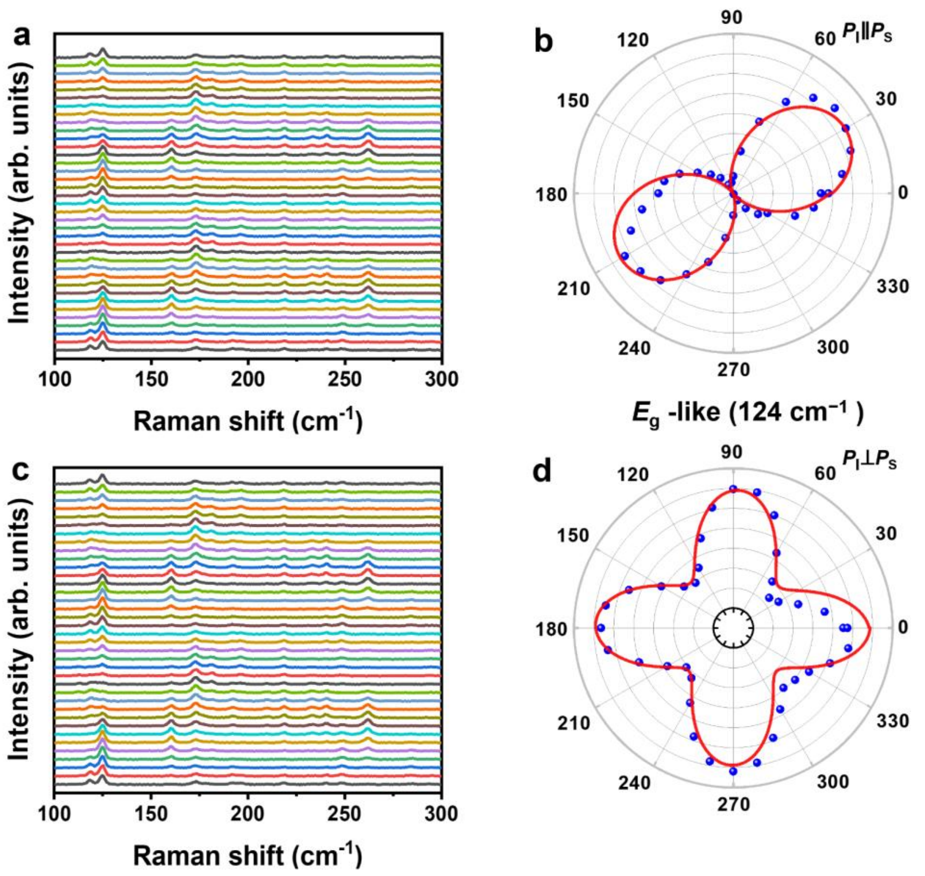

Supplementary Fig. 3 a, c Raman spectra of ~2.5 nm-thick $ReSe_2$ flake at different polarization angles under the "parallel" and "perpendicular" configuration. **b, d** Polar plots of angular-dependent Raman intensity at 124 cm^{-1} in "parallel" and "perpendicular" configuration.

The excitation angle-resolved Raman spectra of ~ 2.5 nm-thick $ReSe_2$ flake under parallel ($P_I \| P_S$) and perpendicular ($P_I \perp P_S$) polarization configurations were measured in Fig. S3a, c, and the intensity of each Raman peak varies significantly with the excitation angle, where P_I and P_S represent the polarization of the incident and scattered light, respectively. The Raman peak of 124 cm^{-1} in the parallel (Fig. S3b) and perpendicular (Fig. S3d) configurations shows distinct periodic variations with polarization angle, thus confirming the anisotropy of the $ReSe_2$ flakes.

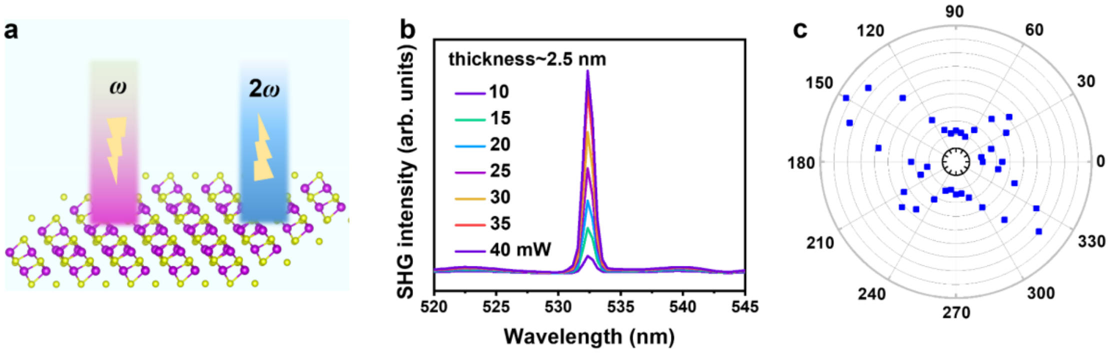

Supplementary Fig. 4 a Schematic diagram of SHG. **b** Excitation power-dependent SHG intensity of ~2.5 nm $ReSe_2$ flake. **c** Polar plots of SHG intensity as a function of polarization angle.

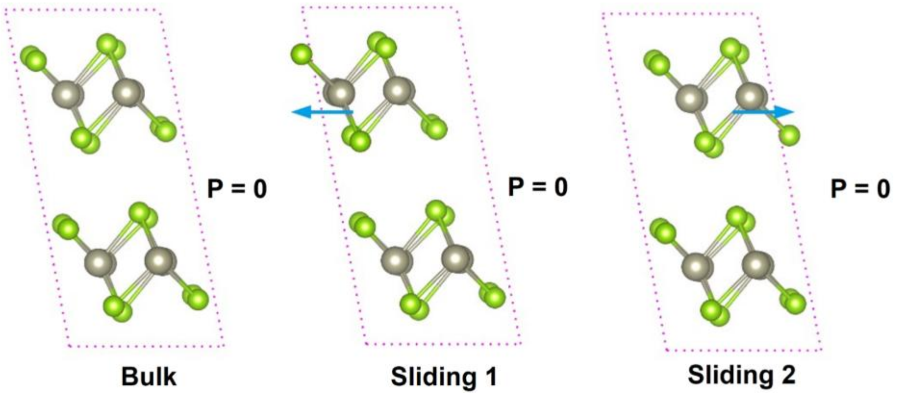

Supplementary Fig. 5 Atomic configuration of interlayer sliding in bilayer $ReSe_2$ system. Interlayer sliding cannot induce ferroelectric polarization due to its spatial inversion symmetry.

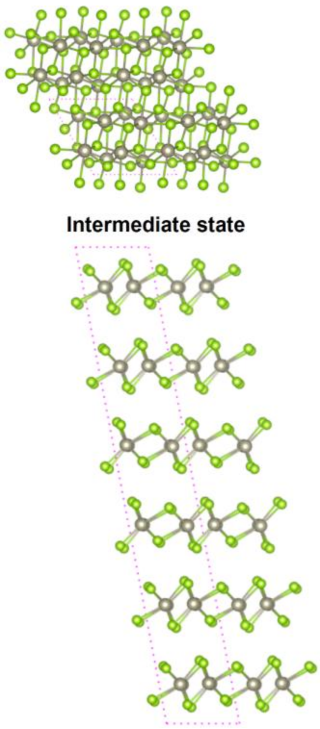

Supplementary Fig. 6 Schematic of atomic arrangement of intermediate state in six-layer $ReSe_2$.

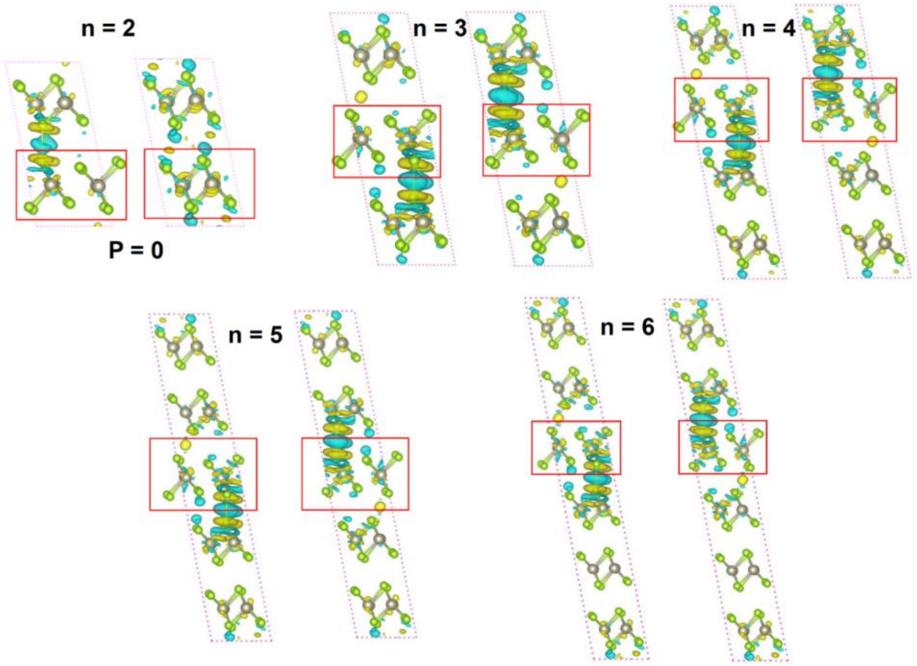

Supplementary Fig. 7 The charge density difference of the sliding one layer in different-layer $ReSe_2$ systems. A net charge transfer occurs in three- or more-layers systems.

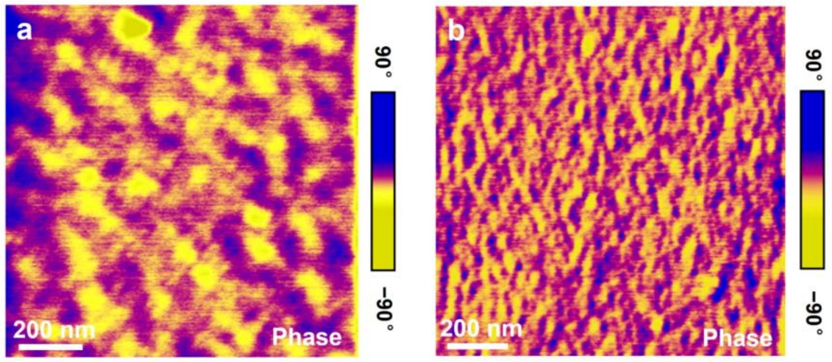

Supplementary Fig. 8 a, b Local out-of-plane PFM phase of Fig. 4a, d.

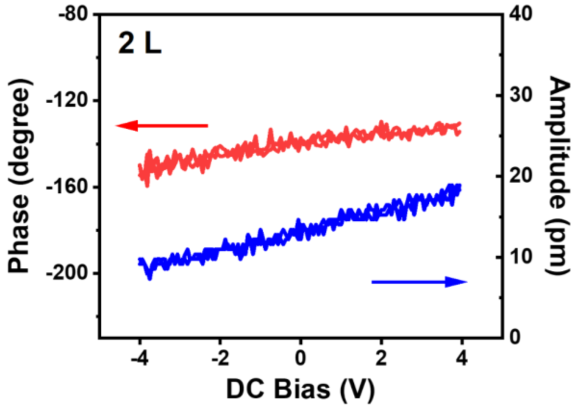

Supplementary Fig. 9 Local PFM phase hysteresis (red) and amplitude (blue) hysteresis loops in two-layer $ReSe_2$.

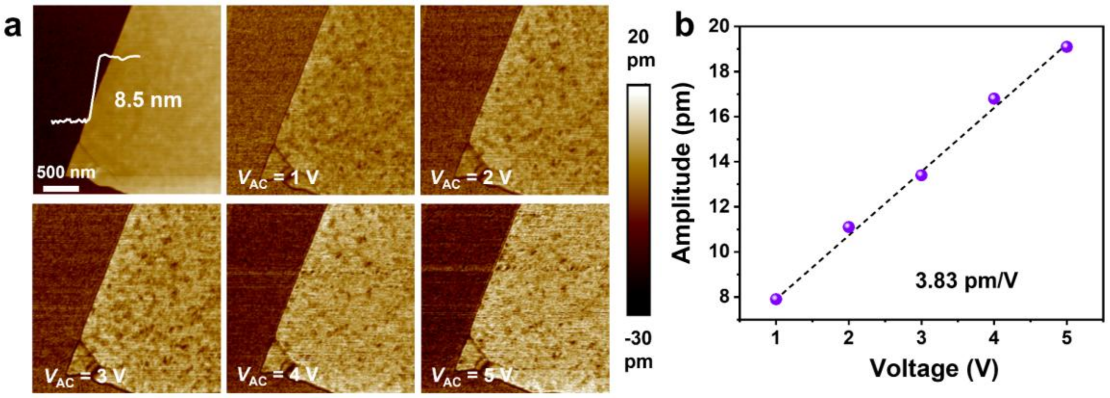

Supplementary Fig. 10 a Height image and OOP amplitude images of 1T' $ReSe_2$ flake under different driving AC voltages. **b** Corresponding OOP amplitude evolution curve with different driving AC voltages.

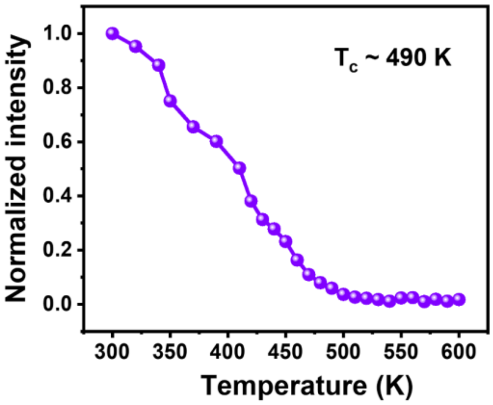

Supplementary Fig. 11 Temperature-dependent of SHG intensity for 1T' $ReSe_2$ (~15 nm), indicating a Curie temperature of approximately 490 K.

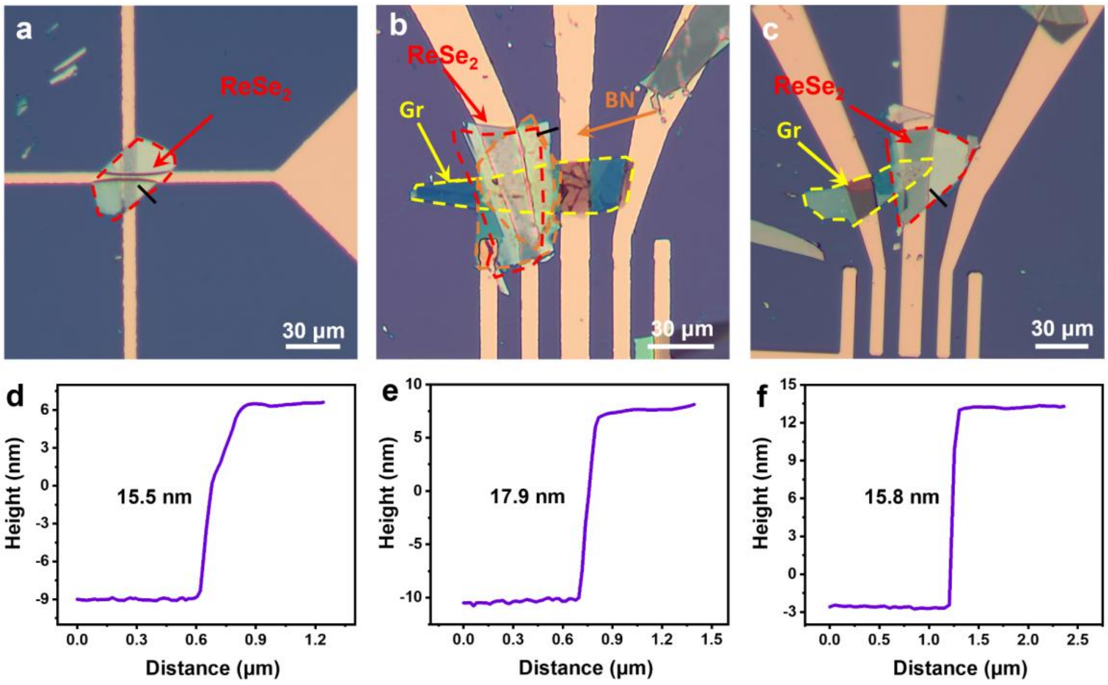

Supplementary Fig. 12 Optical images and thicknesses of $ReSe_2$ for Au/$ReSe_2$/Au (a, d), BN/Gr/$ReSe_2$/BN/Au (b, e) and Gr/$ReSe_2$/Au (c, f) vertical devices.

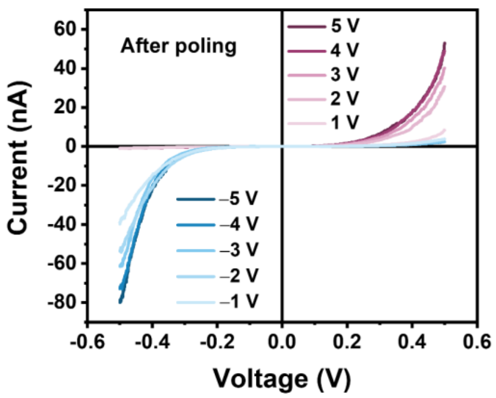

Supplementary Fig. 13 The switchable diode characteristics of $ReSe_2$ device under opposite poling biases.

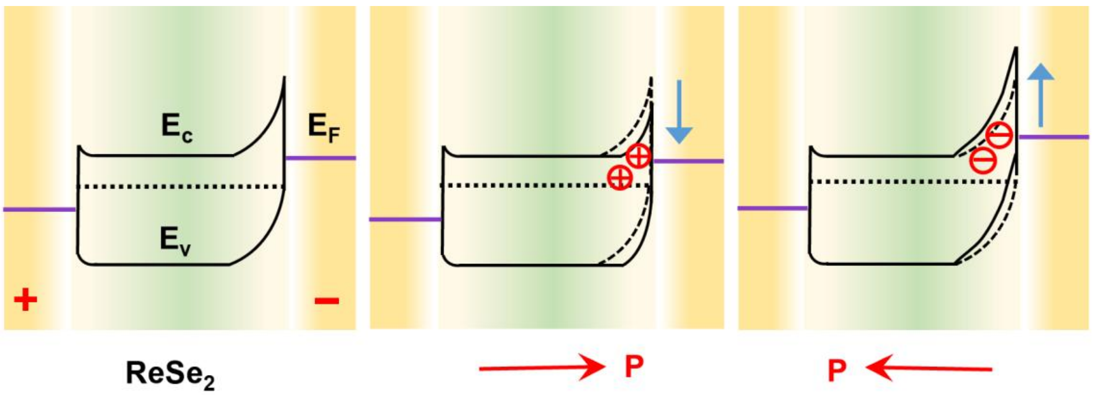

Supplementary Fig. 14 The band diagrams of the $ReSe_2$ ferroelectric diode in the unpoled state and opposite polarized states. The blue arrows indicate the direction of the Schottky barrier change after applying a poling pulse, and red arrows indicate the polarization direction.

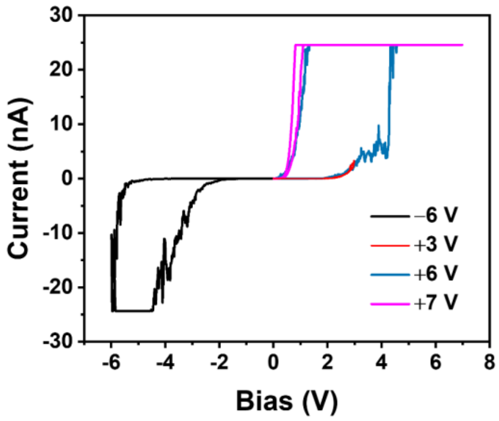

Supplementary Fig. 15 C-AFM *I-V* curves of Pt/1T' $ReSe_2$ (~15 nm)/Pt device at different DC biases.

As shown in Supplementary Fig. 15, the *I-V* loop was significantly open when a −6 V voltage bias was applied, indicating that the sample was polarized to the downward state. Then, three positive voltages of 3 V, 6 V, and 7 V were applied. The results show that the current of the device increases at a 3 V bias and an obvious open loop was formed after applying a 6 V bias with polarization switching to the upward, and the *I-V* loop was not open at a 7 V bias because the upward polarization state has already occurred. This phenomenon is consistent with macroscopic electrical behavior, that is, the partial ferroelectric domains were reversed at a smaller voltage, while a larger bias can provide sufficient driving force to switch more domains.

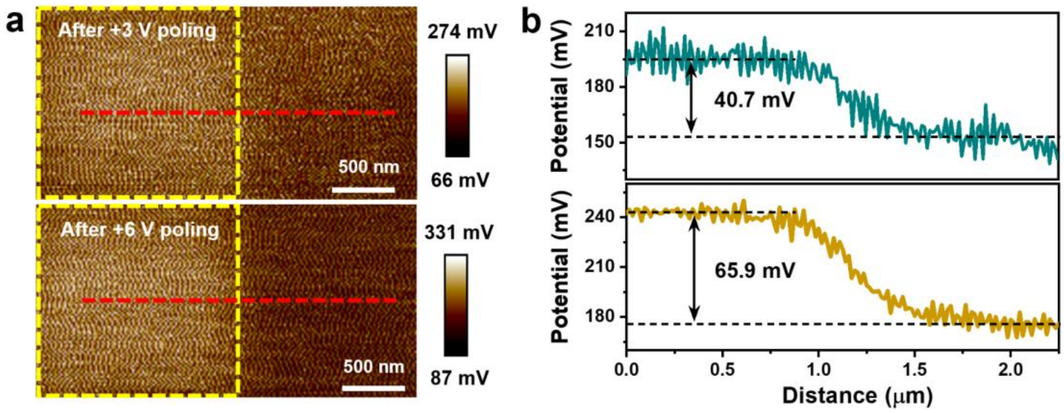

Supplementary Fig. 16 a Surface potential images of 1T' $ReSe_2$ after +3 V and +6 V poling. The yellow dashed box indicates the area of applying voltage. **b** The change of surface potential acquired from the red dashed line in **a**.

KPFM measurements of $ReSe_2$ nanoflake were conducted after applying positive 3 V and 6 V polarization voltages (Supplementary Fig. 16). The results reveal that the surface potential of the polarized region is significantly increased compared to the unpolarized region, and the potential difference reaches 40.7 mV (3 V) and 65.9 mV (6 V), respectively. A higher polarized electric field can more sufficiently drive ferroelectric polarization reversal, resulting in generating a larger potential difference. The voltage dependence of the ferroelectric polarization exhibited in the above nanoscale electrical properties is consistent with the macroscale electrical properties.

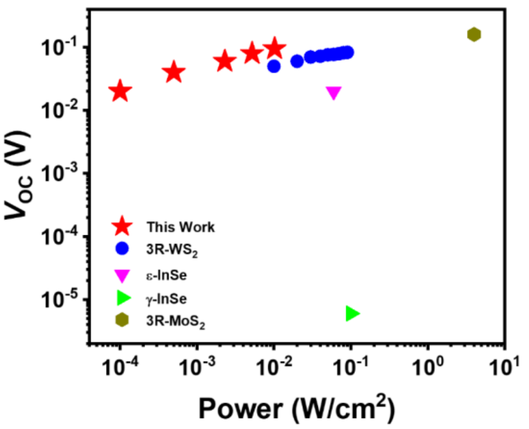

Supplementary Fig. 17 V_{oc} dependent on optical power density in 1T' $ReSe_2$ ferroelectric photovoltaic device and other reported sliding ferroelectric photovoltaic devices[1-4].

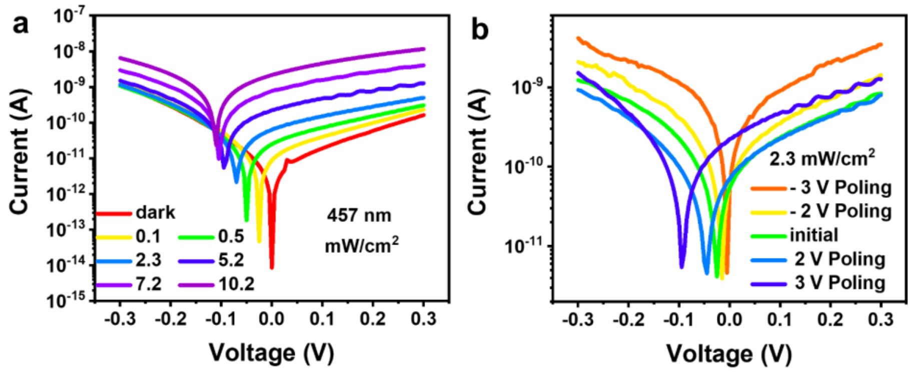

Supplementary Fig. 18 a *I-V* curves of another device in the dark and under different light intensities with a wavelength of 457 nm. **b** Programmable photovoltaic effect after applying poling voltages of 0 V (initial), −2 V, −3 V, 2 V, and 3 V, respectively.

Supplementary references

1. Jingda Wu et al. Ultrafast response of spontaneous photovoltaic effectin 3R-MoS_2–based heterostructures. *Science Advance* **8,** eade3759 (2022).
2. Gong, Y. et al. Reconfigurable and nonvolatile ferroelectric bulk photovoltaics

based on 3R-WS_2 for machine vision. *Nat. Commun.* **16,** 230 (2025).

3. Liang, Q., Zheng, G., Fan, S., Yang, L. & Zheng, S. Multidirectional sliding ferroelectricity of rhombohedral-stacked InSe for reconfigurable photovoltaics and imaging applications. *Adv. Mater.* **37,** 2416117 (2025).

4. Wang, Y. et al. Sliding ferroelectricity induced ultrafast switchable photovoltaic response in ε-InSe layers. *Adv. Mater.* **36,** 2410696 (2024).
