# Supplementary material for: Emergence of sliding ferroelectricity in naturally parallel-stacked multilayer ReSe2 semiconductor
Source: Nat Commun. 2025 Jul 9;16:6313. doi: 10.1038/s41467-025-61756-4 (PMC12238609; doi:10.1038/s41467-025-61756-4)
Supplement: Supplementary file 2 — Laser Reporting Summary [file 41467_2025_61756_MOESM2_ESM.pdf]

nature research

Corresponding author(s): Xiaohong Xu, Shaobo Cheng, Ri He

Lasing Reporting Summary

Nature Research wishes to improve the reproducibility of the work that we publish. This form is intended for publication with all accepted papers reporting claims of lasing and provides structure for consistency and transparency in reporting. Some list items might not apply to an individual manuscript, but all fields must be completed for clarity.

For further information on Nature Research policies, including our data availability policy, see Authors & Referees.

Experimental design

Please check: are the following details reported in the manuscript?

1. Threshold

Plots of device output power versus pump power over a wide range of values indicating a clear threshold

- [ ] Yes
- [x] No

This research did not conduct experiments related to pump power.

2. Linewidth narrowing

Plots of spectral power density for the emission at pump powers below, around, and above the lasing threshold, indicating a clear linewidth narrowing at threshold

- [ ] Yes
- [x] No

This research did not conduct experiments related to linewidth narrowing.

Resolution of the spectrometer used to make spectral measurements

- [ ] Yes
- [x] No

The experiments presented in the manuscript did not involve spectral measurements.

3. Coherent emission

Measurements of the coherence and/or polarization of the emission

- [ ] Yes
- [x] No

The experiments presented in the manuscript did not involve coherent emission.

4. Beam spatial profile

Image and/or measurement of the spatial shape and profile of the emission, showing a well-defined beam above threshold

- [ ] Yes
- [x] No

Experiments related to beam were not included in this research.

5. Operating conditions

Description of the laser and pumping conditions
Continuous-wave, pulsed, temperature of operation

- [ ] Yes
- [x] No

This research did not conduct experiments related to laser and pump.

Threshold values provided as density values (e.g. W cm^{-2} or J cm^{-2}) taking into account the area of the device

- [ ] Yes
- [x] No

This research did not related to threshold or density values for lasers and pumps.

6. Alternative explanations

Reasoning as to why alternative explanations have been ruled out as responsible for the emission characteristics
e.g. amplified spontaneous, directional scattering; modification of fluorescence spectrum by the cavity

- [ ] Yes
- [x] No

This research did not related to the emission characteristics.

7. Theoretical analysis

Theoretical analysis that ensures that the experimental values measured are realistic and reasonable
e.g. laser threshold, linewidth, cavity gain-loss, efficiency

- [ ] Yes
- [x] No

This research did not related to the theoretical analysis of lasing.

8. Statistics

Number of devices fabricated and tested

- [ ] Yes
- [x] No

This research did not related to the devices on lasing.

Statistical analysis of the device performance and lifetime (time to failure)

- [ ] Yes
- [x] No

This research did not related to the devices on lasing.

March 2018
